# Assessment of efficacy and impact on work productivity and attendance after a mandatory switch to generic second-generation antihistamines: results of a patient survey in Norway

**DOI:** 10.1186/1476-7961-9-5

**Published:** 2011-02-28

**Authors:** Fredrik Thorn, Halvor Celius, Tone Ødegård, Randeep Mandla, Erik Hexeberg

**Affiliations:** 1 Nordstrand Legesenter, Oslo, Norway; 2 MSD Norge AS, Drammen, Norway; 3 Dr Hexeberg's Clinic, Sandvika, Norway

## Abstract

**Background:**

In 2006, the Norwegian Medicines Agency mandated a switch from desloratadine, ebastine, or fexofenadine to cetirizine or loratadine in patients with allergic rhinitis (AR) or chronic urticaria (CU). In an online survey, patients whose medication was switched assessed the impact on efficacy, fatigue, and work productivity/attendance.

**Methods:**

Allergy patients in Norway completed a 25-item online survey. Patients aged ≥ 18 years with AR or CU who were switched to cetirizine or loratadine from desloratadine, ebastine, or fexofenadine were included. Participants rated post-switch efficacy, fatigue, and effect on work productivity/attendance compared with their pre-switch medication. Patients also reported post-switch change in number of doctor visits required, total treatment cost, and whether they had switched or wanted to switch back to their previous medications.

**Results:**

Of 1920 patients invited, 493 responded and 409 of these were eligible. Previous antihistamines were desloratadine (78.4% of respondents), ebastine (16.0%), and fexofenadine (5.6%). Post-switch, 64.7% received cetirizine and 35.3% loratadine. Compared with previous therapy, cetirizine and loratadine were rated less effective by 46.3% of respondents; 28.7% reported increased fatigue; and 31.6% reported decreased work productivity with the generic agents. At the time of the survey, 26% of respondents had switched back to their previous medication.

**Conclusions:**

This is the first survey to assess the impact on patient-reported outcomes of a mandated switch from prescription to generic antihistamines in Norway. The findings suggest that patient response to different antihistamines will vary and that treatment decisions should be individualized for optimal results.

## Background

Allergic rhinitis (AR) and chronic urticaria (CU) are common diseases that disturb sleep and reduce work/school productivity [1,2]. Treatment guidelines recommend second-generation antihistamines, including cetirizine, desloratadine, ebastine, fexofenadine, levocetirizine, and loratadine, as a first-line treatment for AR [1] and CU [2].

Although data on comparative efficacy of second-generation antihistamines are limited, clinical studies demonstrate that patients with AR or CU who fail to respond to one antihistamine may benefit from a switch to another [3-6]. In 2006, based on a report [7] that drew no conclusions regarding efficacy or safety differences among the 6 antihistamines mentioned above, the Norwegian Medicines Agency mandated, as a cost-cutting measure and requirement for continued governmental reimbursement, that health care providers substitute generic cetirizine or loratadine for desloratadine, ebastine, or fexofenadine in their AR and CU patients, irrespective of treatment success or patient satisfaction with their current regimen [8]. Only those patients who failed treatment with both cetirizine and loratadine could switch back to the newer agents [8].

An online survey conducted in 2007 in Norwegian general practice settings evaluated patient experiences regarding efficacy, fatigue, and impact on work productivity and attendance after a mandatory switch from desloratadine, ebastine, or fexofenadine to cetirizine or loratadine.

## Methods

Between January and April 2007, general practice centers across Norway each invited up to 15 patients (minimum 1500 in total) to participate in an online survey. Patients were identified through a data software program (eZearch; Emetra AS, Bergen, Norway) run on the physicians' computers. If the practice had more than 15 eligible patients, the total number was randomly reduced to a maximum of 15.

These patients were sent a letter, signed by their physician, inviting them to answer an Internet-based questionnaire. The letter contained a unique username and password that permitted the patients to answer the questionnaire only once. Internet service was provided by Questback AS (Oslo, Norway), an independent IT services group.

Participants aged 18 to 65 years treated with desloratadine, ebastine, or fexofenadine, and switched to cetirizine or loratadine after May 1, 2006, completed the 25-question survey on demographics, antihistamine therapy, physician visits, and treatment cost. Anonymity was maintained by Questback AS using a method approved by the Norwegian Data Inspectorate.

Participants rated post-switch efficacy, fatigue, and effect on work productivity and attendance compared with their pre-switch medication using the following descriptors: "much less," "slightly less," "similar," "slightly more," or "much more." Instances of no response were included in "similar." Patients also reported post-switch change in number of doctor visits required (fewer, same, more), total treatment cost (lower, no change, higher), and whether they had switched back or wanted to switch back to their previous medications.

Descriptive analyses were performed on main findings, and responses by patients are given as proportion of patients (%) in Table 1. Subgroup analyses were conducted on diagnosis, pre-switch antihistamines, and post-switch therapy.

**Table 1:** Baseline demographics and clinical characteristics (N = 409)

Sex, n (%)	
Female	271 (66.3%)

Age (y), n	

18-25	35

26-35	67

36-45	101

46-55	122

56-65	84

Diagnosis, %	

Pollen allergy	63.7

Other nasal allergy	15.2

Skin allergy/eczema	6.9

Other	14.3

Pre-switch therapy, %	

Desloratadine	78.4

Ebastine	16.0

Fexofenadine	5.6

Post-switch therapy, %	

Cetirizine	64.7

Loratadine	35.3

## Results

A total of 343 physicians invited 1920 patients to participate. Of 493 patients (25.7%) responding to the invitation, 421 were eligible (ie, patients stating a switch of medication as a result of the new rules for reimbursement). Twelve patients who failed to specify pre-switch or post-switch antihistamine were excluded. The number of participants included in the final assessment totaled 409 (83.0% of responding patients; Table 1).

Pollen allergy was present in 63.7% of patients. Respondents also reported other nasal allergy (15.2%) and skin allergy (6.9%); other allergies comprised the remaining 14.3%. Most patients (78.4%) were taking desloratadine prior to the mandatory switch, followed by ebastine (16.0%) and fexofenadine (5.6%); post-switch, 64.7% of patients received cetirizine and 35.3% loratadine.

**Figure 1:**
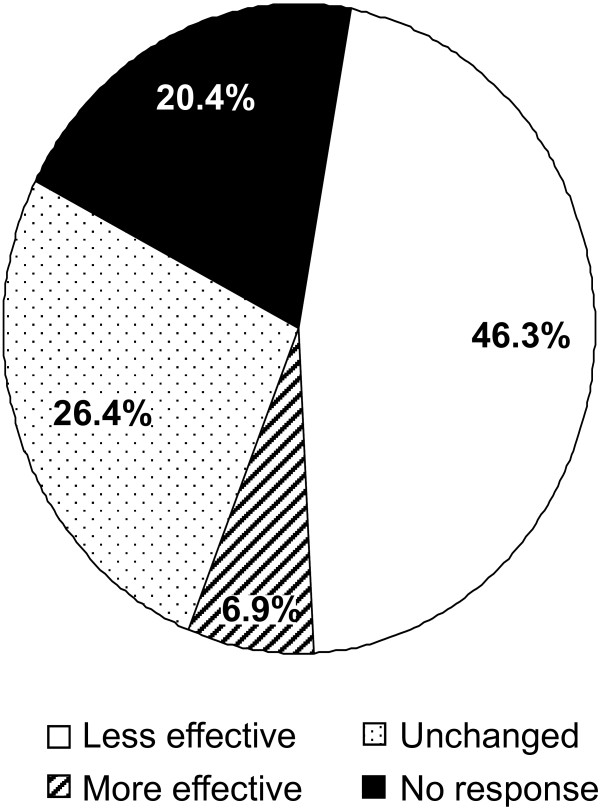
**Post-switch efficacy of cetirizine/loratadine therapy compared with efficacy of desloratadine, ebastine, or fexofenadine pre-switch**.

Respondents (46.3%) rated cetirizine or loratadine as less efficacious than desloratadine, ebastine, or fexofenadine; 21.1% of respondents reported much less efficacy than with their previous treatment; and 25.2% reported slightly less efficacy (Figure 1). In total, 6.9% of respondents rated cetirizine/loratadine more effective than previous treatment (5.0%, slightly more effective; 1.9%, much more effective). Further, 28.7% of respondents reported increased fatigue, while 6.4% were less tired. Another 63.9% reported fatigue levels that were similar between previous and post-switch treatment.

In reporting impact on work productivity, 31.6% of respondents reported decreased work capacity; 63.1% said post-switch work productivity was similar to that of previous treatment, while 3.3% reported greater work productivity after switch. Post-switch, 6.0% reported reduced work attendance, and 2.0% stated that work attendance had increased.

Respondents reported increased contact with their physician post-switch compared with pre-switch: 13% visited their physician more often, compared with 4% who reported fewer visits. Total costs post-switch increased for 16% of respondents and decreased for 10%. Due to limited data, the actual number of consultations and total costs were not possible to measure.

Subgroup analyses stratified by diagnosis, pre-switch antihistamine, or post-switch antihistamine found no significant difference in ratings for efficacy, fatigue, or work productivity and attendance irrespective of type of allergy. This confirmed that the above-mentioned factors had no impact on the outcomes assessed.

**Figure 2:**
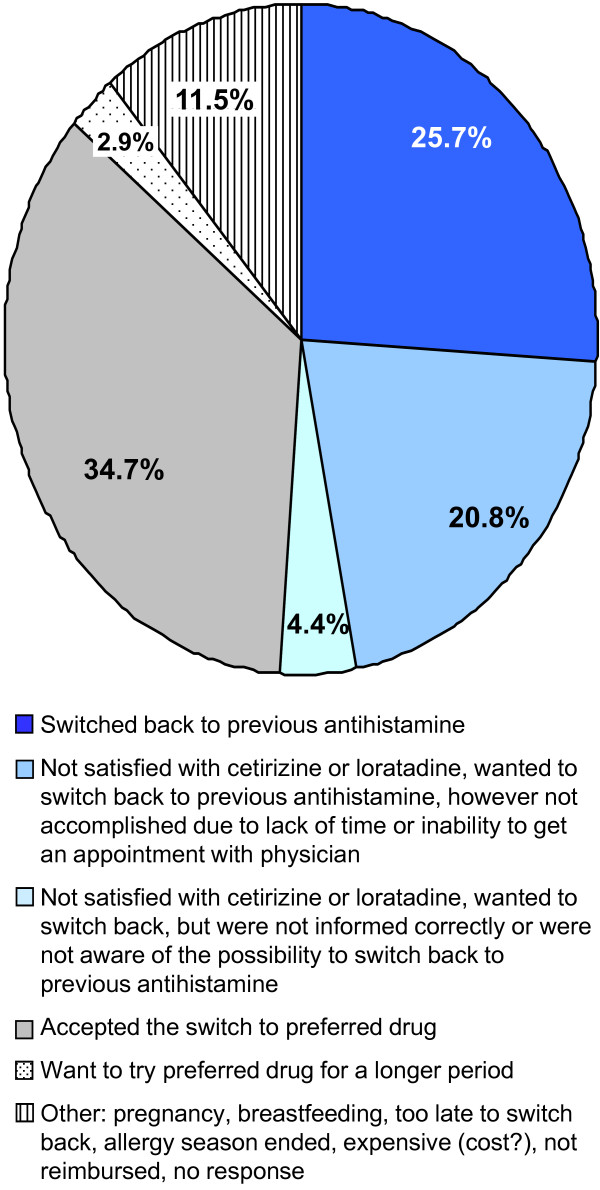
Responses from patients reporting less efficacy post-switch (n = 191)

At the time of the survey, 26% of all participants had already switched back to their pre-switch antihistamine, and 25% reported dissatisfaction with cetirizine or loratadine and wanted to switch back to their original agent; 38% remained on their post-switch agent (Figure 2). Of 191 participants who rated cetirizine/loratadine less effective than desloratadine, ebastine, or fexofenadine, 37% had switched back to their pre-switch antihistamine; an additional 42% reported they were dissatisfied with the mandatory switch in agents and were interested in switching back to their original prescription; 11% remained on their post-switch agent. Further, 63% of respondents reporting less efficacy, more somnolence, or less productivity at work (n = 267) with cetirizine or loratadine were already receiving their previous treatment or wanted to switch back.

## Discussion

This online survey provided the first patient assessment of the impact of a mandatory switch from one antihistamine to another. Although the Norwegian Health Agency claimed that all second-generation antihistamines were equally efficacious and safe [8], 46.3% of respondents perceived reduced efficacy with cetirizine/loratadine compared with their previous drug, 28.7% reported increased fatigue, and 31.6% experienced decreased work productivity.

Patients with AR and CU report varying degrees of symptom relief with different second-generation antihistamines, raising the issue of whether a mandated switch in treatment is ethically acceptable in patients well controlled by or satisfied with their treatment. Clinical management guidelines for urticaria recommend changing to another second-generation antihistamine in patients whose symptoms do not respond to first-choice treatment at standard or higher-than-indicated doses after a defined period [2].

Moreover, clinical studies and patient surveys indicate that switching patients dissatisfied with their current treatment to another second-generation antihistamine may provide symptom relief. In a post hoc analysis of data from 4 post-marketing studies in patients with AR or CU who had failed previous therapy with cetirizine, fexofenadine, or loratadine, 90.3% of patients rated efficacy with desloratadine as excellent or good [3]. In another study, patient satisfaction with CU treatment rose from 74.3% to 83.7% after their antihistamine therapy was modified [4]. A cross-sectional survey of AR patients dissatisfied with loratadine reported equal or better satisfaction after a switch to desloratadine or fexofenadine [5]. Those patients with severe symptoms inadequately controlled by loratadine also reported greater satisfaction with desloratadine compared with fexofenadine. Finally, a randomized, multicenter study found that more patients with AR reported moderate, marked, or complete relief of symptoms after switching to loratadine subsequent to fexofenadine failure than after switching to fexofenadine following loratadine failure [6].

The Norwegian Health Agency conducted no patient-impact assessment or health-economic analysis prior to mandating use of generic antihistamines to maintain governmental reimbursement. Instead, it based its decision solely on the cost of the 6 antihistamines. Post-switch, respondents reported a trend toward more consultations with their physicians, increased total costs for medication, and decreased work productivity and attendance, suggesting that the mandate may not be as cost-effective as assumed.

Some limitations of this survey should be noted. There was no control group; responses were sought only from patients for whom a switch from desloratadine, ebastine, or fexofenadine to loratadine or cetirizine was mandated. In addition, differences in efficacy and tolerability among agents can only be determined through head-to-head, controlled clinical trials. The results of this survey cannot be extrapolated to other groups of allergy patients, and any drug comparisons should be made with great caution.

## Conclusions

A mandatory switch to the generic second-generation antihistamines cetirizine or loratadine to continue reimbursement in patients well controlled on desloratadine, ebastine, or fexofenadine decreased symptom control and work productivity and attendance. Taken together, the results from this survey and from clinical trials and other surveys indicate that treatment decisions should be made only after a thorough patient evaluation and then individualized for each patient. Further, periodic follow-up should be made to assess patient response post-switch.

## Competing interests

Medical writing and editorial assistance was provided by Carol Sibley and Patricia C. Abramo of AdelphiEden Health Communications, New York, New York. This assistance was funded by Merck, Sharpe and Dohme & Co. and MSD Norway AS.

## Authors' contributions

HC, TO and EH conceived the survey and participated in its design and coordination; they also participated in the preparation of the manuscript. FT participated in the design and coordination and had patients who answered the survey. RM performed the statistical analysis and participated in the preparation of the manuscript. All authors read and approved the final manuscript.
